# A New Control Strategy for High-Pressure Homogenization to Improve the Safety of Injectable Lipid Emulsions

**DOI:** 10.3390/pharmaceutics14081603

**Published:** 2022-07-31

**Authors:** Carsten Grumbach, Volker Krüger, Peter Czermak

**Affiliations:** 1 Institute of Bioprocess Engineering and Pharmaceutical Technology, Technische Hochschule Mittelhessen, University of Applied Sciences, Wiesenstrasse 14, 35390 Giessen, Germany; carsten.grumbach@gmail.com; 2 Faculty of Biology and Chemistry, Justus-Liebig-University of Giessen, Heinrich-Buff-Ring 17-19, 35392 Giessen, Germany; 3 B. Braun SE, Carl-Braun-Strasse 1, 34212 Melsungen, Germany; volker.krueger@bbraun.com

**Keywords:** parenteral fat emulsions, injectable lipid emulsions, nano-emulsions, high-pressure homogenizer, counter-jet valve, microfluidizer

## Abstract

Intravenous lipid emulsions are biocompatible formulations used as clinical nutrition products and lipid-based delivery systems for sparingly soluble drugs. However, the particle-size distribution is associated with risks of embolism. Accordingly, the mean particle diameter (MPD) and particle-distribution tailing (characterized as the pFAT5 value) are critical quality attributes that ensure patient safety. Compliance with the limits stated in the United States Pharmacopoeia is ensured by high-pressure homogenization, the final step of the manufacturing process. The US Food and Drug Administration’s Quality-by-Design approach requires a control strategy based on deep process understanding to ensure that products have a consistent and predefined quality. Here we investigated the process parameters of a jet-valve high-pressure homogenizer, specifically their effect on the MPD, pFAT5 value and droplet count (determined by microscopy) during the production of a Lipofundin MCT/LCT 20% formulation. We provide deep insight into droplet breakup and coalescence behavior when varying the process pressure, emulsion temperature and number of homogenization cycles. We found that high shear forces are not required to reduce the pFAT5 value of the particle distribution. Finally, we derived a control strategy for a rapid and cost-efficient two-cycle process that ensures patient safety over a large control space.

## 1. Introduction

Current trends in pharmaceutics show an increased popularity of “nano” methods for the processing of drug excipients (such as lipids and polymers) into nanomaterials or nano drug-delivery systems. The capability of pharmaceutical nanotechnologies in tailoring components, compositions, inner structure and outer morphologies has rapidly increased to follow the trends in nanosciences [1,2]. The direct top-down nanotechnologies such as high-pressure homogenization with counter-jet valve has great potential to produce safe injectable (parenteral) lipid emulsions. Parenteral lipid emulsions are used for the clinical nutrition of patients with a critical nutritional status who can no longer be supplied via the enteral route. They also serve as carriers or delivery systems for lipophilic drugs that are sparingly soluble in water, whereby the active ingredients are incorporated de novo into the oil phase of the emulsion or added extemporaneously [3]. Oil-in-water (O/W) emulsions are preferred for parenteral administration in modern intensive care settings [4]. Herein, we focus on the product Lipofundin, which contains medium-chain triglycerides (MCTs) derived from coconut oil and conventional long-chain triglycerides (LCTs) derived from soybean oil as the disperse phase [5]. The emulsions typically have a fat content of 10–20%, and contain egg lecithin as an emulsifier, sodium oleate as a co-emulsifier, glycerol as an osmotically active additive for isotonicity, lipid-soluble α-tocopherol as an antioxidant, and water for injection (WFI) as the external phase. Manufacturing must ad here to strict requirements with regard to the equipment, raw materials and process steps. The products must be sterile, pyrogen-free, well tolerated by the patient, and stable during storage [6]. In the case of parenteral emulsions, characterization of the particle distribution is one of the most important quality characteristics. The mean particle diameter (MPD) and size distribution of oil droplets must be monitored using particle analytical methods to ensure that limits stated in the United States Pharmacopoeia (USP) are not exceeded. This is because large particles increase the risk of fat embolism [7].

The manufacturing of injectable emulsions for parenteral use differs significantly from that of dermal and peroral emulsions in order to meet USP requirements for microbiological quality and droplet size [8]. The entire manufacturing process is carried out under a nitrogen atmosphere to protect the polyunsaturated fatty acids and lecithin from oxidation. The process can be divided into four main steps. The first is the separate preparation of the water and oil phases, in which the filter-sterilized starting materials are individually dispersed in separate reactors to dissolve the hydrophilic and lipophilic components. In the second step, the oil and water phases are pumped together into a reactor and emulsified into a coarse pre-emulsion (premix) using a rotor-stator stirrer at a controlled temperature with high speed and shear [9]. The third step is the conversion of the coarse premix into a final nano-emulsion by high-pressure homogenization with a specified process temperature, homogenization pressure, and number of cycles. If extraneous particles enter the product during the manufacturing process, they are removed by filtration without damaging the emulsion in a fourth process step. For this purpose, the pH of the emulsion is adjusted to the desired value before filtration (nominal pore size = 5 µm) and filling into the primary packaging material, which is USP type I glass containers [10], plastic containers [11], or three-chamber bags containing glucose, amino acids and the parenteral fat emulsion as a source of calories for parenteral nutrition [12]. The emulsions are then sterilized with steam (121 °C, 0.2 MPa, 15 min) in a rotary autoclave. Rotation achieves defined and gentle mixing of the contents at a specified angular speed during the sterilization process in order to avoid the formation of fat edges in the containers [13].

**Figure 1:**
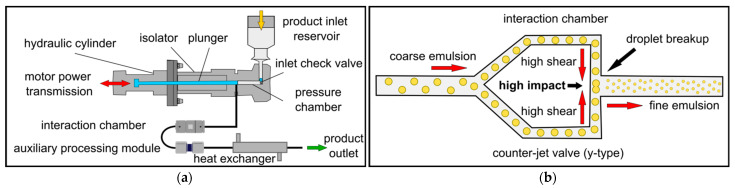
High-pressure homogenizers for the preparation of nanoemulsion products. (**a**) Setup of a jet-valve high-pressure homogenizer consisting of an intensifier pump, interaction chamber, auxiliary processing module and heat exchanger. (**b**) Interaction Y-chamber microchannel, the preferred type for emulsion droplet breakup.

The third process step in the high-pressure homogenizer must ensure the particle size is reduced below the USP threshold [14]. Several high-pressure homogenizers are available, differing in design and geometry mainly with regard to the valve. In dynamic valves (radial diffuser, axial valves), sudden restriction of the flow leads to pressure fluctuations, resulting in products with a wider particle-size distribution [15]. The more recent counter-jet technology features a stainless-steel interaction chamber with adiamond core that is machined with a fixed microchannel structure to deliver constant pressure profiles and narrower particle distributions with smaller particles [16]. To ensure that the high pressure in the interaction chamber does not expand against atmospheric pressure, the flow is stabilized by a downstream auxiliary processing module that builds up counterpressure. Some publications use the term microfluidizer to describe a high-pressure homogenizer with a counter-jet valve. To avoid confusion, we used the term Microfluidizer (capitalized) to describe proprietary machines from the manufacturer Microfluidics, which feature a special counter-jet design with the same mode of operation as a high-pressure homogenizer [17]. Such homogenizing machines essentially consist of a motor that transmits a force to a high-pressure amplifier system consisting of a piston that moves back and forth in a pressure chamber. The piston upstroke draws the premix from a feed hopper into the pressure chamber and the downstroke compresses it to the required homogenizing pressure (Figure 1a). The product is then forced at high pressure through a narrow cross-section of the interaction chamber, where the emulsification process (droplet breakup) takes place. The interaction chamber is the key unit of this technology, and two different types are available. The preferred type for emulsion droplet breakup is the Y-chamber, which splits the product into two high-velocity streams that are accelerated in the interaction chamber and then reunited (Figure 1b). The meeting of the two product streams generates high shear and cavitation forces causing droplet breakup [18]. Immediately before the collision of the streams, the microchannels become narrow, increasing the shear and enhancing the disruption effect. The second type is the Z-chamber, where the product flow is forced through a zigzag microchannel changing the flow direction several times. This promotes particle collision, making such chambers more suitable for the reduction of particle size in suspensions. The product then runs through a heat exchanger for cooling and can be removed via a product hose [19].

The Quality-by-Design approach ensures a deep understanding of control and manufacturing processes based on risk and quality management. This integrates quality during the early stages of product and process development to ensure that the desired product quality can be controlled in ongoing production and that faults can be detected and rectified at an early stage. Comprehensive process understanding requires a precise definition and detailed knowledge of the sequence of production, the most influential and thus the most critical process parameters, their interactions with each other, and their effects on product quality, thus helping to define the design space [20]. A control strategy is necessary for the entire process to ensure that the products have a consistent and predefined quality. Current process understanding of high-pressure homogenization for the preparation of nano-emulsion products focuses on physicochemical properties such as particle size, surface charge, and particle-size distribution [21]. The physicochemical properties of lipid emulsions can be optimized to ensure efficient drug release [22]. The droplet diameter has an impact on the parameters influencing lipoprotein metabolism, capture by the mononuclear phagocyte system, and elimination from the circulation after parenteral application [23]. For parenteral use, safety is associated with the MPD and the large-diameter tail of the drop-size distribution, including the potential for unstable emulsions and the formation of large droplets that can induce embolisms. The pFAT5 value is currently the only parameter used to determine the safety and stability of emulsions, and is defined as the percentage of fat found in globules larger than 5 µm [24].

Herein, we investigated the process parameters (number of cycles, process pressure and emulsion temperature) of a jet-valve high-pressure homogenizer and their effect on the MPD, pFAT5 value and droplet count. We measured the MPD and tailing of the particle distribution by photon correlation spectroscopy (PCS), single-particle optical sensing (SPOS) and droplet counting (microscopy). We then derived a manufacturing process control strategy that ensures patient safety over a large control space.

## 2. Materials and Methods

### 2.1. Lipofundin MCT/LCT 20%

A 10-L batch of the fat emulsion Lipofundin MCT/LCT 20% was produced according to the recipe shown in Table 1.

### 2.2. Emulsification Process

**Figure 2:**
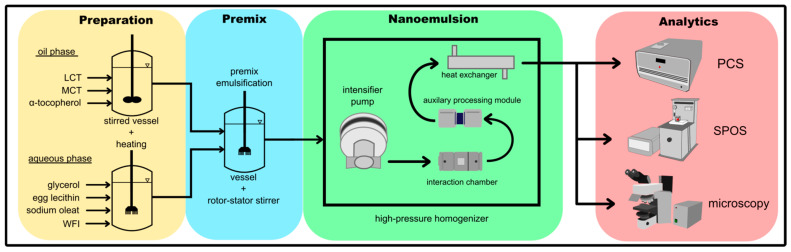
Manufacturing process for parenteral fat emulsions and subsequent analytics. Production starts with the preparation of the aqueous and oil phases, followed by coarse and then fine emulsification in the interaction chamber of a high-pressure homogenizer with an auxiliary processing module and heat exchanger. The product is then characterized by photon correlation spectroscopy (PCS), single-particle optical sensing (SPOS), and microscopy (droplet counts).

The production process consisted of three steps, all carried out under a protective nitrogen atmosphere in a PIC class C cleanroom (Figure 2). In the first step, the water phase (glycerol, sodium oleate and WFI) was placed in a 10-L stirred tank reactor (outer Ø = 315 mm, inner Ø = 240 mm, height (overall) = 640 mm, height (inside) = 480 mm; Lenz Laborglas, Wertheim, Germany) and brought to 65 °C using a Frigomix S cooling water bath with a Thermomix S immersion circulator heater (B. Braun Melsungen, Melsungen, Germany). Egg lecithin was added and dispersed for 60 min at 65 °C on a T 50 digital ULTRA-TURRAX rotor-stator stirrer (IKA-Werke, Staufen im Breisgau, Germany). The soybean oil (LCT) and coconut oil (MCT) were mixed with α-tocopherol in a beaker, and this oil phase was then brought to 65 °C on a hot plate with an IKAMAG RCT magnetic stirrer (IKA-Werke). In the second step, the premix was prepared by adding the oil phase to the water phase in the stirred tank reactor and the mixture was emulsified for 25 min on the rotor-stator stirrer. In the third step, the final fine emulsion was prepared in a modified PSI-40 counter-jet high-pressure homogenizer (PSI Instruments, Pomezia, Italy). The machine was designed to use a 75-µm double-slot interaction chamber with a double-head intensifier-pump. It is an electrically-driven pump system designed to achieve a pressure of up to 2000 bar and throughput of up to 70 L/h. However, the pump and machine control were modified to accommodate a 75-µm single-slot interaction chamber type E101D (PSI Instruments, Pomezia, Italy). To reduce the flow rate to ~20 L/h, the control system was modified so that only one pump head of the double-head pump generated the operating pressure. The premix emulsion was heated on a hot plate or cooled to the appropriate temperature in an ice bath. The premix was then emulsified by recirculating through the high-pressure homogenizer. After each passage, the temperature was adjusted as stated above.

### 2.3. Analytical Methods for Emulsion Characterization

#### 2.3.1. Photon Correlation Spectroscopy

The MPD was determined by PCS using a PCS 380 DLS Nicomb device (Particle Sizing Systems) [11]. We transferred 2–5 µL from the sample bottle to a cuvette using a piston-operated pipette, added 4 mL WFI and mixed to achieve optical homogeneity. The cuvette was then placed in the beam-path of the PCS device and the intensity was recorded at 300 ± 20 kHz. If this was not possible, the sample was either diluted with WFI (intensity > 300 kHz) or more sample was added (intensity << 300 kHz) and homogenized as above. The measurement parameters are listed in Table 2.

#### 2.3.2. Single-Particle Optical Sensing

The fraction volume pFAT5 was determined by single-particle optical sensing (SPOS) using an AccuSizer 780 APS (Particle Sizing Systems) [25]. We transferred ~40 mL from the sample to a 50-mL beaker with a magnetic stirring bar and homogenized at medium speed. The sample tube of the SPOS device was then immersed in the sample. After inputting the percentage volume fraction of the disperse phase and the dilution factor (DF2), automatic measurement commenced. First, the device flushed a 1-mL sample loop over the sample hose. The sample volume from the loop was then pre-diluted (DF1) in a stirred pre-dilution chamber. After the second dilution in the flow-through static dilution unit (DF2), the sample was passed through the flow-through cell into the detector for 4 min. The set volume flow during measurement was 60 mL/min (240 mL of diluted sample per measurement). The output was the total number of counted oil drops per measurement as well as the number of drops in the range 5–50 µm diameter, allowing the pFAT5 to be calculated. The total number of particles (oil drops) counted per measurement should be 100,000–150,000 for correct determination. The dilution factor of samples outside this range was adjusted and the measurement was repeated. The measurement parameters are listed in Table 3.

#### 2.3.3. Optical Microscopy

Particles were counted using a BX51 microscope fitted with a UC30 digital camera (Olympus Deutschland) [26]. We placed ~10 µL of sample in the middle of a clean slide and applied a coverslip to distribute the sample evenly and avoid air pockets. A drop of immersion oil was then placed on the coverslip and the preparation was viewed as a digital image with an oil immersion lens (×100). Four images were captured in the corner areas and one in the middle of the slide. Oil droplets ≥ 2 µm were measured and classified using Stream Enterprise v1.4. (Olympus Corporation, Tokyo, Japan)

#### 2.3.4. Data Analysis

One batch of premix was prepared on each test day. The conditions for preparing the premix were always kept constant (see Section 2.2). The premix was then further processed in the high-pressure homogenizer at the various process conditions. One sample was taken per pass through the high-pressure homogenizer and then measured using the analytical methods described above.

## 3. Results and Discussion

### 3.1. Effect of the Homogenization Cycles

**Figure 3:**
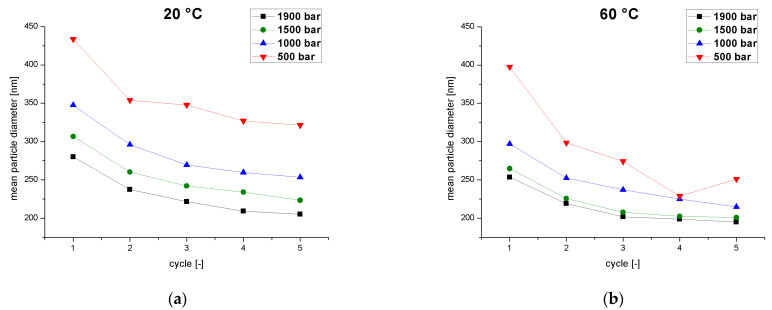
The mean particle diameter as a function of the number of cycles at different operating pressures (500–1900 bar) and two temperatures. (**a**) Emulsion temperature at the inlet of the homogenizer = 20 °C. (**b**) Emulsion temperature at the inlet of the homogenizer = 60 °C.

We initially considered the influences of the operating pressure and the emulsion temperature on the MPD as a function of the number of cycles. The emulsion temperature was always set and measured before passing the sample through the homogenizer. We measured the particle size and the change in particle size between cycles. The target particle size distribution for high-pressure homogenization is given in USP Chapter 729 (Globule Size Distribution in Lipid Injectable Emulsions) and the required MPD is ≤500 nm [11]. We found that the MPD decreased continually over five homogenization cycles, and was already below the 500 nm threshold after the first pass (Figure 3). For each combination of pressure and temperature, the MPD fell from cycle to cycle to a minimum value, in agreement with the plateau effect reported by others [27,28,29]. This phenomenon reflects the non-uniform shear in the interaction chamber. Droplets passing through the chamber near the walls experience less shear than droplets in the center of the flow. Multiple passes through the chamber equate to a higher residence time and, therefore, increase the probability of individual droplets passing through the high-shear zones of the chamber [27]. Accordingly, the number of cycles is directly related to the MPD at a constant pressure and temperature. We observed an interaction between the number of passes and the homogenization pressure and also the premix temperature. A lower MPD was associated with higher temperatures (Figure 3a) and higher pressures (Figure 3b) because both factors promote droplet breakup. High temperatures decrease the viscosity and surface tension of the emulsion where as high pressures increase the energy input [17]. In summary, higher temperatures and pressures require fewer cycles to produce small MPDs and also reduce the influence of the number of cycles on the MPD.

Particle-size reduction decreased from cycle to cycle within the pressure range 1000–1900 bar and the temperature range 20–60 °C. The viscosity of the emulsion decreases from cycle to cycle caused by irreversible changes in rheological properties due to elastification [30]. Lower viscosities shorten the residence time of the emulsion and lower the energy input in the interaction chamber to limit MPD reduction from cycle to cycle [31]. The effect of MPD reduction, which is calculated from the difference in MPD from the previous and subsequent cycle, was stronger at low pressures, especially during the first pass. This pressure-dependent effect on MPD reduction can be explained by the fact that the MPD reduction is smaller if a high energy input into the emulsion has already occurred during the previous high-pressure cycle. Each further cycle thus has a smaller effect on MPD reduction. Such behavior has been investigated by the repeated homogenization of a silicone O/W nano-emulsion using a counter-jet homogenizer [32]. The authors defined a saturation radius above which no further reduction in droplet size occurs, which was dependent on operating pressure and viscosity. The MPD approaches this saturation radius over successive cycles, resulting in a saturation curve [32].

The MPD reduction was very strong during the first cycle at low pressure and high temperature. We recorded the highest MPD reduction of 99 nm (397.5 nm–298.5 nm = 99 nm) at 500 bar and 60 °C from the first to the second circle. At higher pressures (1000–1900 bar) the MDP reduction decreases. Temperature also loses its influence on MPD reduction at higher pressures, resulting in greater MPD reduction at high pressure and low temperature (20 °C). This is because we approach the saturation radius faster with fewer passes at higher temperatures and pressures, and the effect on MPD reduction decreases faster. An exception occurs from the fourth cycle at a pressure of 1000 bar, where this effect is reversed at 60 °C. Here, the higher temperature results in a smaller saturation radius, so more droplets are broken up and there is a greater reduction of MPD. At a lower pressure of 500 bar, a temperature of 60 °C consistently shows a greater effect on the MPD compared to 20 °C. Furthermore, at a pressure of 500 bar, the MPD reduction alternates between strong and weak effects in contiguous cycles. Such coalescence effects are seen at high temperatures (60 °C) and low temperatures (20 °C), which cause the oscillating reduction of MPD. The coalescence effect is stronger at high temperatures, even causing the MPD to increase in the fifth cycle. This reflects the weakdroplet-breakup forces at low pressures due to the low energy input, so the coalescence effects limit the reduction in MPD. The alternating droplet breakup and coalescence forces do not achieve the saturation radius after five passes. A dynamic equilibrium may exist between droplet breakup and coalescence in the interaction chamber. The complex temperature effects and the lower pressure combined with the inlet MPD of the emulsion passing through the interaction chamber leads to a shift in the equilibrium towards droplet breakup or coalescence in an alternating manner.

Various mechanisms of coalescence are described in the literature [33]. For the operating pressure of 500 bar and low temperature of 20 °C, we observed clogging of the interaction chamber during processing. This suggests mechanisms such as droplet aggregation or partial coalescence, which increase the viscosity due to the aggregation of partially crystalline oil droplets [34]. The higher the fat volume fraction of an emulsion, the more it tends to partial coalescence [34]. The fat crystals are formed in the core of the oil droplets. When the fat droplet is deformed by shear, the crystals can cause local structural changes on the droplet surface that promote aggregation [35]. For the operating pressure of 500 bar and high temperature of 60 °C, we observed stronger coalescence effects which increase the MPD during the fifth pass. Given that significantly smaller droplet sizes are achieved with the Lipofundin MCT/LCT 20% formulation, coalescence cannot be attributed to a lack of emulsifier. This overprocessing effect is frequently reported [31] as a critical point of processing, and has been attributed to several factors [36,37,38,39]. Many authors attribute the effect to an insufficient emulsifier concentration resulting in the incomplete saturation of the particle surface, or have observed coalescence mainly when higher pressure causes a higher energy input [33] or when the cycle number increases [40]. Others attribute this effect to increased Brownian motion promoting collision and coalescence [41]. Tech and Schubert defined the coalescence frequency as the product of the collision frequency and the probability of coalescence [42]. This, in turn, depends on the dispersed phase concentration, viscosity, energy input, and droplet diameter. The MPD increases rapidly in line with the coalescence frequency. According to this dependency, a lower emulsion viscosity, a larger MPD, and a higher energy input increase the collision frequency and probability of coalescence. Experiments at higher process pressures show no coalescence-dominated MPD reduction and the emulsifier has no effect at the given process temperature, so we attribute the increase in coalescence and overprocessing effects to the MPD, viscosity ratio, surface tension and flow conditions at the selected process temperature and pressure. Both the temperature and number of cycles affect the viscosity of the emulsion. If the viscosity ratio increases, coalescence is favored and the MPD also increases. Depending on the viscosity, droplet-breakup mechanisms change from laminar to turbulent inertial and turbulent viscous [43]. MPD reduction thus strongly depends on the emulsion quality during the previous pass, as well as the temperature and pressure. The effect is strongest during the first pass and decreases from cycle to cycle [44]. The highest MPD reduction of 99 nm was observed during the first pass at low pressure (500 bar) and high temperatures (60 °C), whereas the smallest MPD (194.9 nm) was measured at a pressure of 1900 bar and 60 °C in the fifth cycle. At a process pressure of 500 bar, there is a clear coalescence effect that causesthe MPD to fluctuate.

**Figure 4:**
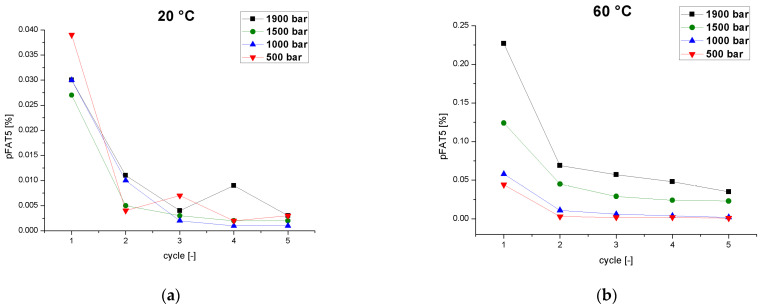
The pFAT5 value as a function of the number of cycles at different operating pressures (500–1900 bar) and two different temperatures. (**a**) Emulsion temperature at the inlet of the homogenizer = 20 °C. (**b**) Emulsion temperature at the inlet of the homogenizer = 60 °C.

The pFAT5 threshold is specified as ≤0.05% in USP Chapter 729 (Globule Size Distribution in Lipid Injectable Emulsions) [24].A study of the preparation of a soybean oil nano-emulsion using a counter-jet valve high-pressure homogenizer at 1000–1200 bar and 25 °C showed no significant decrease in the pFAT5 value (0.01–0.02%) from cycles 6 to 10, indicating that six passes is sufficient for a safe and stable nano-emulsion [45]. Therefore, we tested the effect of the number of cycles on the tailing of the droplet distribution (Figure 4). The low process temperature of 20 °C was associated with significantly lower pFAT5 values than the same process at 60 °C, and fell within the acceptable range after the first cycle. Low temperatures, therefore, reduce the number of cycles required to achieve a pFAT5 value < 0.05%. ThepFAT5 value also showed a significant dependence on pressure with increasing temperature. At 60 °C and a homogenization pressure of 1900 bar, the pFAT5 value fell below the limit value for the first time after the fourth cycle. At pressures of 1500 and 1000 bar, the limit was achieved after only two cycles. At 500 bar, only a single cycle was required even at 60 °C. This contrasts with a previous study in which the pFAT5 value of a similar formulation decreased with increasing pressure, with pressure and cycle number proposed as the most important influencing variables [46]. However, the tests were carried out in a high-pressure homogenizer with a dynamic valve and a temperature below 40 °C, and different emulsifiers were used (cholesterol, soybean lecithin, and poloxamer 188).

The first pass through a high-pressure homogenizer has the greatest effect on the pFAT5 value but the effect decreases with increasing temperature [44]. Here, we observed the fluctuation ofpFAT5 values at 500 bar with an increasing number of cycles. As discussed above, these fluctuations represent partial coalescence effects caused bychamber clogging, which are more pronounced at 500 bar and 20 °C due to the significantly larger MPD with crystal nucleation. This effect counteracts droplet breakup, thereby influencing the pFAT5 value. The effect was not observed at 500 bar and 60 °C (Figure 4). Low temperatures and low pressures greatly reduce tailing in the distribution and one or two passes were sufficient under these conditions to achieve an acceptable pFAT5 value. The number of cycles was in influenced by the emulsion temperature as well as the process pressure. Especially at high temperatures, the pFAT5 value showedclear dependence on pressure and tended toward a pressure-dependent minimum value. At low pressure and temperature, the residence time in the chamber was longer, giving the droplets sufficient time to deform as they passed through the chamber. In addition, the vortices that form in the turbulent field last longer than the time required for the droplets to deform and break up [13]. The time required for droplet breakup increases with droplet diameter [14]. Longer residence times in the chamber increase the probability that larger droplets are deformed and broken up, thus reducing the pFAT5 value.

**Figure 5:**
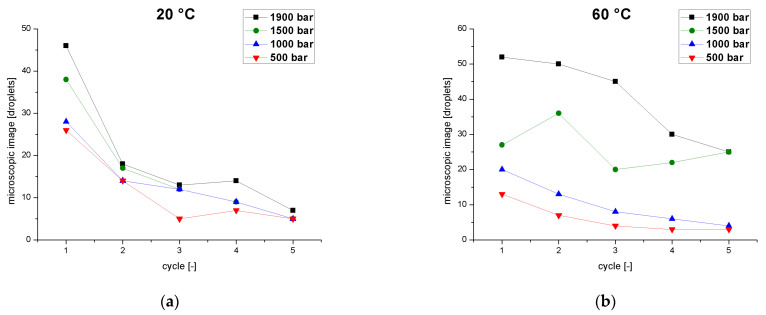
Droplet count determined by microscopy as a function of the number of cycles at different operating pressures (500–1900 bar) and two different temperatures. (**a**) Emulsion temperature at the inlet of the homogenizer = 20 °C. (**b**) Emulsion temperature at the inlet of the homogenizer = 60 °C.

Droplet counts based on microscopy provided further information about the tailing of the particle distribution (Figure 5). The total number of droplets was counted for each individual pass through the homogenizer as a function of pressure at two process temperatures. At 20 °C (Figure 5a), the number of droplets was influenced slightly by the pressure but showed a strong dependence on the number of passes. The first passage showed a strong decline in the number of counted drops, and although further reduction was observed in each subsequent pass, the effect was smaller and tended toward a plateau at the minimum value. As described for the pFAT5 values, fewer droplets were detected at lower pressures. At 60 °C (Figure 5b), we observed the same behavior at low pressures of 500–1000 bar, but different behavior emerged at higher pressures. At 1900 bar, the number of droplets decreased from cycle to cycle with a similar magnitude until the fourth cycle, with a smaller decline in the fifth cycle. At 1500 bar, the droplet number initially increased from the first to the second cycle, but then fell again in the third cycle before a linear increase in the fourth and fifth cycles. This behavior is caused by the superposition of droplet breakup and coalescence effects.

**Figure 6:**
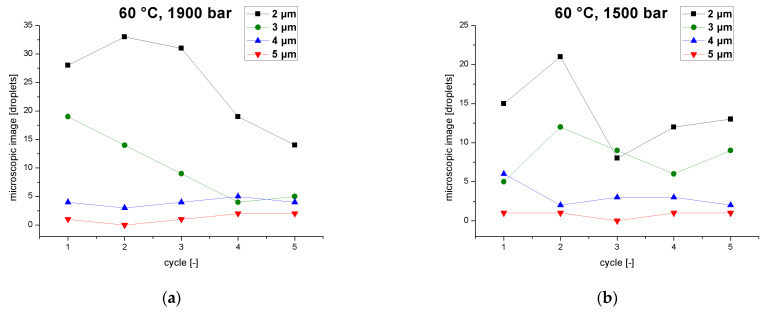
Droplet count of different particle size classes (2–5 µm)in an emulsion as a function of the number of cycles when the inlet temperature of the homogenizer is 60 °C, at operating pressures of (**a**) 1900 bar, and (**b**) 1500 bar.

The viscosity of the emulsion falls from cycle to cycle as the MPD declines with each pass. As the viscosity changes, so does the residence time of the emulsion in the interaction chamber. The droplet count was evaluated as a function of the number of passes in terms of individual particle-size classes for a pressure of 1900 bar and a temperature of 60 °C, revealing that the emulsion is processed differently from cycle to cycle (Figure 6a). During the first pass, particles of 3–5 µm are broken up, increasing the abundance of smaller particles (2 µm). From the second to the fourth pass, droplet breakup shifts to smaller particles of 2–3 µm, whereas larger particles of 4–5 µm accumulate due to recoalescence effects. Smaller particles are broken up, with the loss of 2-µm particles increasing from the second to the fourth cycle. During the fifth pass, degradation shifts to particles ≤ 2 µm. Large particles of 3–5 µm show no change or even an increase. The abundance of smaller particles (2 µm) reaches a plateau. The higher temperatures of 60 °C combined with high pressures lead to more droplets due to the coalescence effects. This agrees with a previous study based on a 10% sunflower-oil emulsion, which found that smaller droplet sizes with narrower distribution can be produced in jet-valve homogenizer by cooling [47].

Figure 6b shows the droplet count (individual particle size classes) as a function of the number of passes at a process pressure of 1500 bar and an emulsion temperature of 60 °C. The droplet count is lower overall than at 1900 bar but changes very slightly over the five cycles from 27 droplets in the first pass to 36, 20, 22 and25 droplets in the fifth pass. This reflects the balance between breakup and coalescence under these process parameters. During the first pass, droplets of 4 µm are broken up, whereas smaller droplets coalesce. This effect is reversed during the second pass, where small droplets (2–3 µm) break up and large droplets (4–5 µm) show overlapping breakup and coalescence effects. After the third pass, small droplets (2–3 µm) also show overlapping breakup and coalescence effects, where as large particles show only coalescence effects. During the fifth pass, small particles show only coalescence effects, whereas large droplets breakup. These alternating effects influencing all four particle sizes cause the droplet count to vary between 20 and 36 droplets over the five cycles.

### 3.2. Effect of Temperature and Pressure

**Figure 7:**
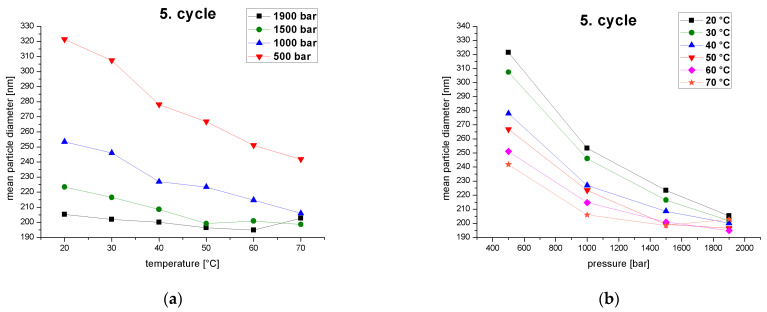
MPD of the fifth homogenization cycle as a function of (**a**) temperature at different operating pressures (500–1900 bar) and (**b**) operating pressure at different emulsion temperatures at the inlet of the homogenizer (20–70 °C).

High temperatures and pressures caused a stronger droplet breakup effect and thus led to a smaller MPD (Figure 7). This agrees with previous studies reporting a significant decrease in the droplet size at higher homogenizer temperatures and pressures [48,49]. The MPD decreased with increasing temperature at constant pressure. The effect of reducing the MPD was greater at lower pressures, as indicated by the larger negative slope of the curves (Figure 7a). The effect of temperature on the reduction of the MPD therefore declines as the pressure increases. The MPD also fell with increasing pressure at constant temperature. The effect was greater at lower temperatures, again as indicated by the larger negative slope of the curves (Figure 7b). The effect of pressure on the reduction of the MPD therefore declines with increasing temperature. This clearly demonstrates an interaction between temperature and pressure, but pressure exerts the greater influence. A study on the optimization of a palmoil nano-emulsion prepared using a counter-jet valve high-pressure homogenizer developed a response surface model of the process, revealing that pressure had a much stronger effect on the MPD compared to the number of cycles and the emulsifier concentration [25].

High temperatures and pressures act additively to generate stronger droplet-breakup effects and thus lead to smaller MPDs. In addition, both effects run into a plateau. Higher temperatures reduce the viscosity of the continuous and disperse phases as well as the interfacial tension and Laplace pressure, promoting droplet breakup with a lower energy input and shear intensity [50]. High pressure increases shear intensity and induces turbulent flow conditions [51]. A simultaneous increase in pressure and temperature, therefore, promote droplet breakup, resulting in smaller MPDs [52]. However, the shear forces generated by the pressure do not lead to smaller MPDs when the pressure is increased further, but reach a minimum value due to the limited quantity of emulsifier. High pressure also increases the collision frequency, which promotes coalescence [48].

**Figure 8:**
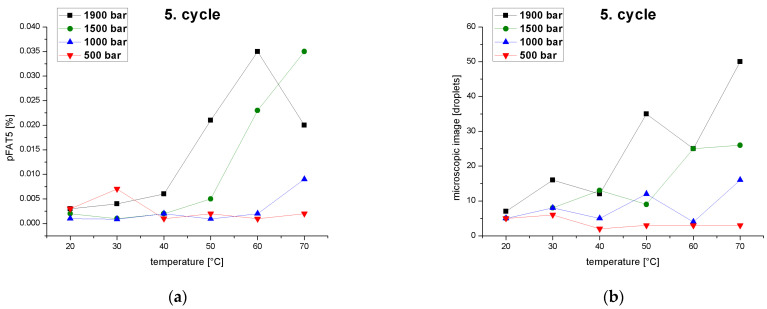
Effect of temperature and pressure on the pFAT5 value and droplet counts. (**a**) The pFAT5 value of the fifth homogenization cycle as a function of temperature at different operating pressures (500–1900 bar). (**b**) Droplet count as a function of temperature at different operating pressures (500–1900 bar).

At constant pressure, the tailing of the droplet-size distribution (pFAT5 value) increased when a certain temperature was exceeded because coalescence effects overcame droplet-breakup effects, but if the temperature continued to rise the effect reversed (Figure 8a). At 500 bar, the effect was already relatively strongly at 30 °C and continued with slight fluctuations. At 1000 bar, the effect first appeared (relatively weakly) at 40 °C, followed by fluctuations and then reappearance (more strongly) from 70 °C upward. At 1500 bar, the effect occurred weakly from 30 °C, then continued to increase. Above 60 °C, the pFAT5 value increased even further, but the effect flattened out. At 1900 bar, the effect occurred from 30 to 60 °C, but was overlaid by the droplet breakup from 60 °C, which reduced the pFAT5 value again. Coalescence effects can therefore occur from 30 °C and lead to a significant increase in the pFAT5 value at high pressures (1500–1900 bar). Over the entire series of experiments, low pressures correlated with the smallest pFAT5 values at temperatures of 40–60 °C, suggesting that high shear forces are unnecessary to reduce the pFAT5 value. High residence times in the interaction chamber and temperatures exceeding 40 °C are ideal for particularly small pFAT5 values. A similar formulation prepared using a counter-jet valve high-pressure homogenizer was optimized using a central composite design, and the operating pressure was the strongest model term with a significantly stronger effect on the pFAT5 value than the number cycles or emulsifier concentration [53]. The sharp drop in the pFAT5 value within a homogenization pressure range of 455–827 bar and a further decrease increasing homogenization pressure was attributed to coalescence effects induced by the high energy input [53].

The behavior of the pFAT5 values was also confirmed by droplet counting following the fifth pass through the high-pressure homogenizer at constant pressures as a function of temperature (Figure 8b). All curves showed an increase in the droplet count with rising temperatures, again reflecting the superposition of coalescence and droplet breakup effects. This confirms that low temperatures and pressures reduce droplet formation and thus tailing.

### 3.3. Derived Control Strategy

Based on the data set out above, we developed a strategy to control the MPD independently from the tailing of the particle distribution. To facilitate the production of injectable lipid emulsions in the shortest possible time and at the lowest possible cost, the control strategy was based on a two-cycle process. The MPD was controlled mainly during the first pass by freely selecting the pressure and temperature. The pFAT5 value and droplet count were controlled mainly during the second cycle and were reduced to a level that is safe for the patient.

Table 4 shows the MPD of the second cycle at different process temperatures (20–70 °C). The control space for the MPD spans a range of 229.4–363.4 nm. All MPDs in this range can be produced within two cycles by controlling the pressure (500–1900 bar) and temperature (30–60 °C).

**Figure 9:**
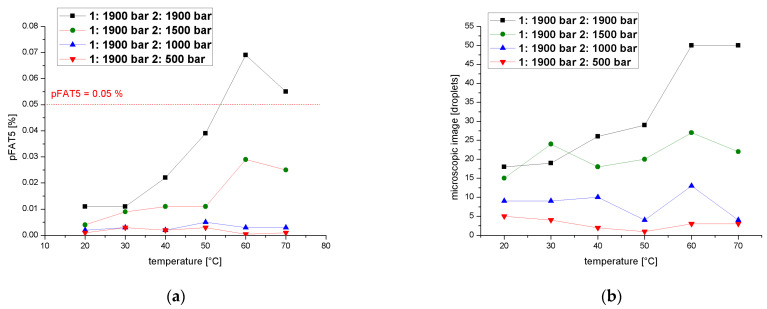
Effect of a two-cycle process (first cycle =1900 bar) on the pFAT5 value and droplet counts. (**a**) The pFAT5 value of the second homogenization cycle as a function of temperature at different operating pressures (500–1900 bar). (**b**) The droplet count of the second homogenization cycle as a function of temperature at different operating pressures (500–1900 bar).

We determined pFAT5 values after two cycles over a temperature range of 20–70 °C at a process pressure of 1900 bar during the first cycle and 1900, 1500, 1000 or 500 bar in the second, revealing that the pFAT5 value was reduced most significantly during the second cycle at 500 bar regardless of the temperature (Figure 9a). At 500 bar, the droplet count followed a similar profile (Figure 9b) and did not vary by more than five droplets throughout the temperature range. A comparison of droplet counts between the emulsions homogenized in the first cycle at 1900 bar and in the second cycle at 1900, 1500 and 1000 bar shows that the second cycle is temperature-dependent (Figure 9b). The temperature dependence increases with increasing pressure and exceeds the limit for the pFAT5 value of 0.05% at higher pressures of 1900 bar.

**Figure 10:**
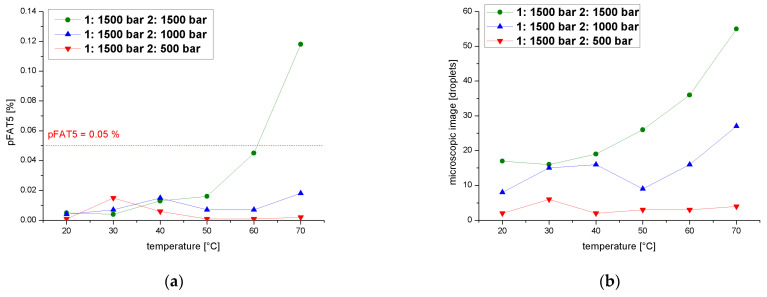
Effect of a two-cycle process with the first cycle at 1500 bar on the pFAT5 value and droplet counts. (**a**) The pFAT5 value of the second homogenization cycle as a function of temperature at different operating pressures (500–1500 bar). (**b**) The droplet count of the second homogenization cycle as a function of temperature at different operating pressures (500–1500 bar).

We also determined pFAT5 values after two cycles over a temperature range of 20–70 °C at a process pressure of 1500 bar during the first cycle and 1900, 1500, 1000 or 500 bar in the second, revealing again that the pFAT5 value was reduced most significantly during the second cycle at 500 bar (Figure 10a). The curve shows a slight increase in pFAT5 value for the temperature range 30–40 °C compared to the other temperatures, caused by coalescence effects, but this still falls within an acceptable range (0.015% at 30 °C and 0.006% at 40 °C). The droplet count followed a similar profile (Figure 10b) and did not vary by more than six droplets throughout the temperature range.

**Figure 11:**
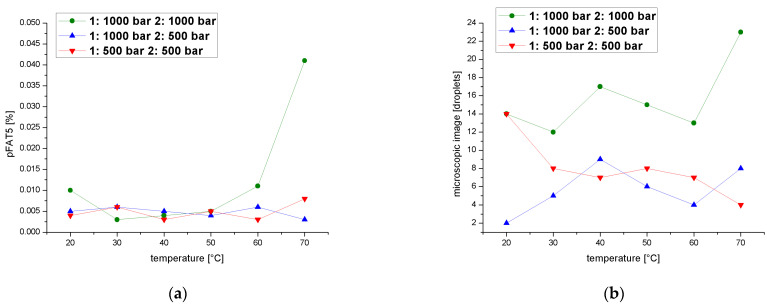
Effect of a two-cycle process with the first cycle at 500 or 1000 bar on the pFAT5 value and droplet counts. (**a**) The pFAT5 value of the second homogenization cycle as a function of temperature at different operating pressures (500–1000 bar). (**b**) The droplet count of the second homogenization cycle as a function of temperature at different operating pressures (500–1000 bar).

Finally, we determined pFAT5 values after two cycles over a temperature range of 20–70 °C at a process pressure of 1000 or 500 bar in each cycle. The pFAT5 value was temperature-independent when the second cycle was set to 500 bar, regardless of the pressure in the first cycle, but when both cycles were set to 1000 bar, we observed temperature dependence that resulted in higher pFAT5 values at 20 and 70 °C, although the value never exceeded0.008% (Figure 11a). A comparison of droplet counts between the emulsions homogenized in the first cycle at 1000 bar and in the second cycle at 1000 and 500 bar shows that the second cycle at 500 bar clearly reduces the droplet count, whereas the second cycle at 1000 bar is temperature-dependent (Figure 11b). The emulsion homogenized in the second cycle at 500 bar mainly shows a droplet count below nine, with the exception of a single value of 14 at 20 °C when both cycles are carried out 500 bar.

## 4. Conclusions

We found that high-pressure homogenization with a counter-jet valve has the potential to produce safe injectable lipid emulsions below the threshold values of MPD and pFAT5, as required by the USP. We investigated the influence of the number of cycles, the emulsion temperature, and the homogenization pressure on the MPD and the tailing of the emulsion particle distribution.

We found that the first pass was the most effective at reducing the MPD and pFAT5 value. Two cycles were sufficient to ensure the MPD and pFAT5 values were well below the USP-specified limits. The homogenization pressure had the greatest influence, strongly reducing the MPD. Increasing the number of cycles and increasing the temperature also reduced the MPD, but to a lesser extent. The process temperature had the greatest influence on the tailing of the particle distribution (increasing the pFAT5value) but this influence declined at low pressures (500 bar). Increasing the pressure also moderately reduced thepFAT5 value whereas increasing the number of cycles had a weak reducing effect. The number of cycles had the strongest influence on the droplet count determined by microscopy, with more cycles strongly reducing the droplet counts, but this effect was opposed by increasing the pressure. A low pressure (500 bar) reduced the number of droplets in the distribution tailing. High shear forces are not required to reduce the tailing of the particle distribution. A pressure of 500 bar led to a strong reduction of the pFAT5 value. Coalescence at 500 bar affected the MPD but not the pFAT5 value. The distribution tailing was affected by coalescence at higher pressures.

Based on these findings, we developed a control strategy that allows the MPD to be controlled largely independently of the pFAT5 value. This process can be carried out with only two cycles at different pressures, the first to control the MPD and the second to reduce tailing and thus the pFAT5 value. The optimized settings are shown in Table 5.This enables the rapid production of emulsions by brief high-pressure homogenization, which also provides more flexibility with regard to the particle size in the final product, thus ensuring product stability and patient safety.

## 5. Patents

The work described in this manuscript is declared in patent no.DE102018205493A1.

## Figures and Tables

**Table 1:** Lipofundin MCT/LCT 20% recipe and raw material suppliers.

Raw Material	Amount	Supplier
Glycerol	250.0 g	August Hedinger
Egg lecithin	120.0 g	Lipoid
Sodium oleate	3.0 g	Sigma-Aldrich Chemie
Alpha-tocopherol	2.0 g	Brenntag
Refined Soybean Oil IV	1000.0 g	Gustav Heess
Medium chain triglyceride	1000.0 g	Cremer Oleo
Water for injection	7625.0 g	B. Braun Melsungen

**Table 2:** Measurement parameters on the PCS 380 DLS Nicomb device.

Measurement Parameter	Setting
Light source	He-Nelaser (632.8 nm)
Angle	90° (singe angle analysis)
Channel width	Automatic (20–30 µs)
Temperature	23 °C
Viscosity (continuous phase)	0.933 cP (water at 23 °C)
Refractive index (continuous phase)	1.333 (water)
Measurement time	10 min
Number of measurements	1

**Table 3:** Measurement parameters on the AccuSizer 780 APS device.

Measurement Parameter	Setting
Data Collection Time	90 s
Number of Channels	128
Diluent Flow Rate	60 mL/min
Target Concentration	1000 Part/mL
Injection Loop Volume	1.0 mL
Syringe Volume	2.5 mL
Initial 2nd-Stage Dilution Factor	100

**Table 4:** Mean particle diameter (MPD) achieved by the two-cycle process at different operating pressures for each cycle and different emulsion temperatures at the inlet of the homogenizer.

Temperature [°C]	MPD [nm] Cycle 1: 1900 Bar Cycle 2: 500 Bar	MPD [nm] Cycle 1: 1500 Bar Cycle 2: 500 Bar	MPD [nm] Cycle 1: 1000 Bar Cycle 2: 500 Bar	MPD [nm] Cycle 1: 500 Bar Cycle 2: 500 Bar
20	273.7 ± 27.1	275.6 ± 89.6	332.6 ± 98.1	353.9 ± 167.7
30	264.6 ± 52.9	270.2 ± 7.8	303.5 ± 27.0	363.4 ± 102.5
40	265.2 ± 52.0	268.9 ± 42.8	305.7 ± 87.1	338.8 ± 72.8
50	255.1 ± 42.9	253.8 ± 47.0	281.8 ± 78.3	313.9 ± 99.8
60	229.4 ±48.9	256.0 ± 14.6	267.0 ± 23.0	298.5 ± 102.4
70	237.8 ± 63.0	251.0 ± 16.3	264.8 ± 33.6	290.5 ± 50.3

**Table 5:** Optimal process variables and their settings as control strategy for the two-cycle process.

Cycle	Pressure	Temperature
primary	500–1900 bar	30–60 °C
secondary	500 bar	30–60 °C

## Data Availability

The data that support the findings of this study are available from the corresponding author upon reasonable request.
